# The Library of the Physician

**Published:** 1894-06-02

**Authors:** 


### The First Century of English Medical Literature.

### IV.—THE LIBRARY OF THE PHYSICIAN.

(*Continued.*)

We have seen that the English library of the Elizabethan physician contained mere fragments of the ancients whom he was still supposed to be able to read fluently in Latin, and was confined to versions of Continental works of varying degrees of merit. The achievements of Paracelsus in the early part of the century had infused fresh vigour into the profession in France and Germany, and had influenced for good those most opposed to his teaching. If only to counteract his heresies physicians were roused to activity, and the echo of the strife in time reached English shores and produced its due effect.

A work written by R. B., Esq., 1585, gives a very graphic description of the controversy raging against the theories of Paracelsus at the time. He calls it "The difference between the ancient physic first taught by the godly fathers, consisting in unity, peace, and concord, and the latter physick proceeding from idolaters, ethnics, and heathen; as Galen and such other, consisting in duality, discord, and contrariety." R. B., Esq., endeavours with great boldness to turn the flank of the enemy and prove that the theories of Paracelsus dated back from Adam, Abraham, and Hermes Trismegestus, and that Galen is a mere modern innovator. The important point in the treatise is his justification of Paracelsus from the charge freely brought against him of being in league with the devil. It is hard to realise how completely medicine was bound up with magic, even to a date far beyond this time. It would seem that Paracelsus set himself vehemently against all "necromancy, sorcery, ceremonies, conjurations, and all manner of invocations of devils, demons, and evil spirits; he saith that the devil cannot cure nor help an ague nor the toothache, and that the devil himself with all his legions hath not so much power or authority that he is able to mar one pot, much less is he able to make him." All this is notably in advance of the beginning of the century when Paracelsus was practising. It is worth comparing with an early Black Letter translation of the "Little Practyce of Johannes de Vigo in Medicine," a noted physician and surgeon in Rome contemporary with Paracelsus. De Vigo enjoyed a great reputation, and was one of the first to achieve success with mercurial friction in skin diseases, and with the use of mercury in small-pox. But what can be said of his specific for "the letarge in the hinder part of the head?" It runs as follows in the translation: "Take a pure black cat and flee her and take out her bowels and pick away the fat from the guts and put it into the body again, and fill the body full of mustard seed well steeped in the juice of neppe and sage, then sew the body up and roast it on a spit till it be dry; then take the droppings and put it in balls, and when you will occupy it, shave the patient in the neck, and annoint him by the fire in the joint next the head and sanabitur." The obvious meaning of the process is a combat with the demon afflicting the patient to be exorcised only by the slaughter and manipulation of the "pure black cat." It is a specimen of a very common manner of prescription, which the example of Paracelsus undoubtedly did much to discredit.

Among the immediate followers of Paracelsus was Joseph du Chesne, commonly known as Quercetanus, who practised medicine and surgery at the court of Henry IV., and died in 1609. His frequent use of antimony prejudiced him in the eyes of later French physicians. His "Practice of Clinicall and Hermetical Physick" was translated into English in 1605 by Thomas Tymme, a minister or divine, and was the most clear exposition of the new medicine which had yet appeared.

A very exhaustive and complete work on medicine was translated in 1598 by James Mose, M.D., from the German of Christopher Wirzung, professor of medicine at Augsburg, who died 1573. The work is intended solely for the use of the physician. It is a large folio volume, entitled "Praxis Medicinæ Universalæ," and professes to treat of all diseases from the crown of the head to the sole of the foot. It was a learned and useful compendium, containing for the most part grave and practicable prescriptions, unsullied by magical elements, and was perhaps the most serviceable work of reference which the times afforded.

"The Boock of Physic," translated from the Dutch of Gaebelkhover in 1599, printed at Dordrecht and dedicated to Queen Elizabeth, is another compendium of a somewhat simpler description. Thus it will be seen that the French, German, Italian, and Dutch contributed to the education of the Elizabethan physician, and were available for him in his own language. Of original English work in medicine there is as yet no sign, for the collected prescriptions of Hester can be dignified by no such name, and other native works, as will be seen later on, were commonly compilations, strung together by laymen for laymen. Christopher Langton, who in 1547 published "The Principal Parts of Physic," added what he called an "Introduction to Physic" in 1550. Both books are made up of references to Galen and Avicen, and seem designed to give the most elementary instruction, possibly to the young student of medicine. Even sixty or seventy years later this sort of general disquisition seems to have passed for learning, as in the "Whole Course of Physic," a curious medley translated by Valentinus, 1612, and in the "Epistle to the Young Student of Physick," by Philiatros, 1615.

The following quaint complaint prefixed to one of these early compilations throws some light on the status of the general practitioner of that time:—

And can a man in gripping grief
  Have aid to ease his smart,
Of such as have no skill at all
  In noble Physic's art?

A retchless rout of doltish dorres
  Have overspread our soil,
Which do bereave the bees also
  Of that whereof they toil.

They trot about from place to place
  As frogs hop in the rain;
Where one or two by hap they heal,
  A hundred more are slain.

